# Supplementary material for: The importance of human factors in therapeutic dietary errors of a hospital: A mixed-methods study
Source: PLoS One. 2022 Aug 25;17(8):e0273728. doi: 10.1371/journal.pone.0273728 (PMC9409594; doi:10.1371/journal.pone.0273728)
Supplement: S1 Questionnaire — (DOCX) [file pone.0273728.s001.docx]

1. **Checklist for Identification of Errors at Various Levels of Food Flow**

|  |  | **Information Checklist for all In-patients regardless of Error** | | | | | | | **Information Checklist for all In-patients with Dietary Errors** | | | |
| --- | --- | --- | --- | --- | --- | --- | --- | --- | --- | --- | --- | --- |
| **S.No** | **Date** | **Diet Error (Y/N)** | **Day Of Week** | **Ward ^1^** | **Diagnosed**  **Disease** | **Meal ^2^** | **NPO**  **(Y/N)** | **Type of Diet ^3^** | **Point of Error ^4^** | **Responsible Staff ^5^** | **Patient Length of Stay** | **Type of Error (Critical / Non-Critical)** |
| **1** | / /2019 |  |  |  |  |  |  |  |  |  |  |  |
| **2** | / /2019 |  |  |  |  |  |  |  |  |  |  |  |
| **3** | / /2019 |  |  |  |  |  |  |  |  |  |  |  |
| **4** | / /2019 |  |  |  |  |  |  |  |  |  |  |  |
| **5** | / /2019 |  |  |  |  |  |  |  |  |  |  |  |
| **6** | / /2019 |  |  |  |  |  |  |  |  |  |  |  |
| **7** | / /2019 |  |  |  |  |  |  |  |  |  |  |  |
| **8** | / /2019 |  |  |  |  |  |  |  |  |  |  |  |
| **9** | / /2019 |  |  |  |  |  |  |  |  |  |  |  |
| **10** | / /2019 |  |  |  |  |  |  |  |  |  |  |  |
| **11** | / /2019 |  |  |  |  |  |  |  |  |  |  |  |
| **12** | / /2019 |  |  |  |  |  |  |  |  |  |  |  |
| **13** | / /2019 |  |  |  |  |  |  |  |  |  |  |  |
| **14** | / /2019 |  |  |  |  |  |  |  |  |  |  |  |
| **15** | / /2019 |  |  |  |  |  |  |  |  |  |  |  |
| **16** | / /2019 |  |  |  |  |  |  |  |  |  |  |  |
| **17** | / /2019 |  |  |  |  |  |  |  |  |  |  |  |
| **18** | / /2019 |  |  |  |  |  |  |  |  |  |  |  |
| **19** | / /2019 |  |  |  |  |  |  |  |  |  |  |  |
| **20** | / /2019 |  |  |  |  |  |  |  |  |  |  |  |
| **21** | / /2019 |  |  |  |  |  |  |  |  |  |  |  |
| **22** | / /2019 |  |  |  |  |  |  |  |  |  |  |  |
| **23** | / /2019 |  |  |  |  |  |  |  |  |  |  |  |
| **24** | / /2019 |  |  |  |  |  |  |  |  |  |  |  |
| **25** | / /2019 |  |  |  |  |  |  |  |  |  |  |  |

| 1. **WARDS:**  - Cardiac Care Unit - Kidney Transplant - Medical Ward - Gastro - Orthopedic - Surgical - Neurological | 1. **MEAL:**  - Breakfast - Lunch - Dinner | 1. **TYPE OF DIET:**  - Clear Liquid - Full Liquid - Semi Solid - Soft - Regular | 1. **POINT OF ERROR:**  - Diet Card Preparation - Meal Preparation/Packing - Tray Line - Meal Provision | 1. **Responsible staff**  - Unit Representative(UR) - Dietitian - Cook - Service Aid |
| --- | --- | --- | --- | --- |

***KEYS:***

1. **Semi Structured Questionnaire for the Source of Error**
2. Are you aware that a wrong diet was sent to a patient?

(If the responsible person says “Yes”, then go to Q.2

If the responsible person says “No”, then inform him/her about the error and go to Q.2)

1. In your view, what might be the reason(s) of the error?
2. What could be done to prevent the current error in the future?
