# Supplementary material for: The importance of human factors in therapeutic dietary errors of a hospital: A mixed-methods study
Source: PLoS One. 2022 Aug 25;17(8):e0273728. doi: 10.1371/journal.pone.0273728 (PMC9409594; doi:10.1371/journal.pone.0273728)
Supplement: Attachment — Submitted filename: PONE-D-21-39046.pdf [file pone.0273728.s002.pdf]

PLOS ONE

The importance of human factors in therapeutic dietary errors of a hospital: A mixed-methods study

--Manuscript Draft--

**Manuscript Number:**	PONE-D-21-39046
**Article Type:**	Research Article
**Full Title:**	The importance of human factors in therapeutic dietary errors of a hospital: A mixed-methods study
**Short Title:**	Human Factors Therapeutic Dietary Errors
**Corresponding Author:**	Naveed Sadiq, Ph.D.<br>Khyber Medical University<br>Peshawar, PAKISTAN
**Keywords:**	Dietary Errors, Human Factors, Patient Safety, Mixed-Methods, Food Safety
**Abstract:**	Accurate therapeutic diet aids in improving medical condition of patients. Any discrepancy in this regard may hamper clinical condition of a patient. In this regard, the study was aimed to assess prevalence of dietary errors to in-patients in the food department of international private hospital, and to explore associated causes to suggest strategies to reduce such errors. A sequential explanatory mixed-methods study was conducted in a private hospital of the country's capital. Quantitative data, included observations, was collected over a one-month period using a prescribed proforma. Errors arising during the meal flow process were traced to the origin and source of error, replacing incorrect diet with correct one, followed by qualitative interview with staff responsible. Quantitative data was analyzed in SPSS v.20 as percentages. Qualitative data was analyzed by deductive-inductive thematic analysis. We found that out of 7041 diets, only 17 had errors. Of these, almost two-thirds were critical. Majority of errors took place during diet card preparation (52.94%), by dietitians (70.59%), during weekdays (82.35%), breakfasts (47.06%), and in the cardiac care ward (47.06%). The causes identified through interviews were lack of backup or accessory food staff, and employee's personal and domestic issues. It was concluded that though the prevalence of dietary errors was low in this study, critical errors constituted majority of these errors, which may hamper clinical condition of patient. Adopting organizational behavior principles in hospital, by considering human factors, may not only reduce dietary errors, but improve patient well-being, and employee satisfaction in a long run.
**Order of Authors:**	Amanullah Khan, MPH, BS (Nutrition)
	Sidra Malik, MPH, BDS
	Fayaz Ahmad, PhD, MPH, MBBS
	Naveed Sadiq, Ph.D.
**Additional Information:**	
**Question**	**Response**
**Financial Disclosure**<br><br>Enter a financial disclosure statement that describes the sources of funding for the work included in this submission. Review the submission guidelines for detailed requirements. View published research articles from *PLOS ONE* for specific examples.<br><br>This statement is required for submission	The funders had no role in study design, data collection and analysis, decision to publish, or preparation of the manuscript.

Powered by Editorial Manager® and ProduXion Manager® from Aries Systems Corporation

and **will appear in the published article** if the submission is accepted. Please make sure it is accurate.<br><br>**Unfunded studies**<br>Enter: *The author(s) received no specific funding for this work.*<br><br>**Funded studies**<br>Enter a statement with the following details:<br>• Initials of the authors who received each award<br>• Grant numbers awarded to each author<br>• The full name of each funder<br>• URL of each funder website<br>• Did the sponsors or funders play any role in the study design, data collection and analysis, decision to publish, or preparation of the manuscript?<br>• **NO** - Include this sentence at the end of your statement: *The funders had no role in study design, data collection and analysis, decision to publish, or preparation of the manuscript.*<br>• **YES** - Specify the role(s) played.<br><br>* typeset	
**Competing Interests**<br><br>Use the instructions below to enter a competing interest statement for this submission. On behalf of all authors, disclose any competing interests that could be perceived to bias this work—acknowledging all financial support and any other relevant financial or non-financial competing interests.<br><br>This statement is required for submission and **will appear in the published article** if the submission is accepted. Please make sure it is accurate and that any funding sources listed in your Funding Information later in the submission form are also declared in your Financial Disclosure statement.<br><br>View published research articles from *PLOS ONE* for specific examples.	The authors have declared that no competing interests exist.

Powered by Editorial Manager® and ProduXion Manager® from Aries Systems Corporation

**NO authors have competing interests**<br><br>Enter: *The authors have declared that no competing interests exist.*<br><br>**Authors with competing interests**<br><br>Enter competing interest details beginning with this statement:<br><br>*I have read the journal's policy and the authors of this manuscript have the following competing interests: [insert competing interests here]*<br><br>* typeset	
**Ethics Statement**<br><br>Enter an ethics statement for this submission. This statement is required if the study involved:<br><br>• Human participants<br>• Human specimens or tissue<br>• Vertebrate animals or cephalopods<br>• Vertebrate embryos or tissues<br>• Field research<br><br>Write "N/A" if the submission does not require an ethics statement.<br><br>General guidance is provided below. Consult the submission guidelines for detailed instructions. **Make sure that all information entered here is included in the Methods section of the manuscript.**	The ethical permission to conduct this study was granted by the Advance Studies & Review Board of the Khyber Medical University, Pakistan (No. DIR/KMU-AS&RB/IE/000966 dated 17th January 2020).<br><br>The interviewees were explained the purpose of the interview/research, and subsequently orally consented for the interview(s).

Powered by Editorial Manager® and ProduXion Manager® from Aries Systems Corporation

**Format for specific study types**<br><br>**Human Subject Research (involving human participants and/or tissue)**<br>• Give the name of the institutional review board or ethics committee that approved the study<br>• Include the approval number and/or a statement indicating approval of this research<br>• Indicate the form of consent obtained (written/oral) or the reason that consent was not obtained (e.g. the data were analyzed anonymously)<br><br>**Animal Research (involving vertebrate animals, embryos or tissues)**<br>• Provide the name of the Institutional Animal Care and Use Committee (IACUC) or other relevant ethics board that reviewed the study protocol, and indicate whether they approved this research or granted a formal waiver of ethical approval<br>• Include an approval number if one was obtained<br>• If the study involved *non-human primates*, add *additional details* about animal welfare and steps taken to ameliorate suffering<br>• If anesthesia, euthanasia, or any kind of animal sacrifice is part of the study, include briefly which substances and/or methods were applied<br><br>**Field Research**<br><br>Include the following details if this study involves the collection of plant, animal, or other materials from a natural setting:<br>• Field permit number<br>• Name of the institution or relevant body that granted permission	
**Data Availability**<br><br>Authors are required to make all data underlying the findings described fully available, without restriction, and from the time of publication. PLOS allows rare exceptions to address legal and ethical concerns. See the PLOS Data Policy and FAQ for detailed information.	No - some restrictions will apply

Powered by Editorial Manager® and ProduXion Manager® from Aries Systems Corporation

A Data Availability Statement describing where the data can be found is required at submission. Your answers to this question constitute the Data Availability Statement and **will be published in the article**, if accepted.<br><br>**Important:** Stating 'data available on request from the author' is not sufficient. If your data are only available upon request, select 'No' for the first question and explain your exceptional situation in the text box.<br><br>Do the authors confirm that all data underlying the findings described in their manuscript are fully available without restriction?	
**Describe where the data may be found in full sentences. If you are copying our sample text, replace any instances of XXX with the appropriate details.**<br><br>• If the data are **held or will be held in a public repository**, include URLs, accession numbers or DOIs. If this information will only be available after acceptance, indicate this by ticking the box below. For example: *All XXX files are available from the XXX database (accession number(s) XXX, XXX.*).<br>• If the data are all contained **within the manuscript and/or Supporting Information files**, enter the following: *All relevant data are within the manuscript and its Supporting Information files.*<br>• If neither of these applies but you are able to provide **details of access elsewhere**, with or without limitations, please do so. For example:<br><br>*Data cannot be shared publicly because of [XXX]. Data are available from the XXX Institutional Data Access / Ethics Committee (contact via XXX) for researchers who meet the criteria for access to confidential data.*<br><br>*The data underlying the results presented in the study are available from (include the name of the third party*	Data cannot be shared publicly because it will lead to the identification of hospital personnel through the designations mentioned in the individual observations. Data are available from the Ethics Committee (contact via email) for researchers who meet the criteria for access to confidential data.

and contact information or URL).<br>• This text is appropriate if the data are owned by a third party and authors do not have permission to share the data.<br><br>* typeset	
Additional data availability information:	Tick here if your circumstances are not covered by the questions above and you need the journal’s help to make your data available.

Manuscript
Click here to access/download;Manuscript;Plose One Format.docx

The importance of human factors in therapeutic dietary errors of a hospital: A mixed-methods study

Authors:

Amanullah Khan[1], Sidra Malik[2], Fayaz Ahmad[1], Naveed Sadiq[1]

[1] Institute of Public Health & Social Sciences, Khyber Medical University, Peshawar, Pakistan

[2] Riphah International University. Islamabad, Pakistan

[*]Corresponding Author

Email: naveedsadiq@gmail.com

Authors' Contribution Statement:

AK made substantial contributions to conception and design of, acquisition of data, analysis and interpretation of data; drafted the article, and revised it critically for important intellectual content; and approved the final approval of the version to be published. SM, FA, and NS made substantial contributions to conception and design of data; revised it critically for important intellectual content, and approved the final approval of the version to be published. All the authors agree to be accountable for all aspects of the work in ensuring that questions related to the accuracy or integrity of any part of the work are appropriately investigated and resolved.

Abstract

Accurate therapeutic diet aids in improving medical condition of patients. Any discrepancy in this regard may hamper clinical condition of a patient. In this regard, the study was aimed to assess prevalence of dietary errors to in-patients in the food department of international private hospital, and to explore associated causes to suggest strategies to reduce such errors. A sequential explanatory mixed-methods study was conducted in a private hospital of the country's capital. Quantitative data, included observations, was collected over a one-month period using a prescribed proforma. Errors arising during the meal flow process were traced to the origin and source of error, replacing incorrect diet with correct one, followed by qualitative interview with staff responsible. Quantitative data was analyzed in SPSS v.20 as percentages. Qualitative data was analyzed by deductive-inductive thematic analysis. We found that out of 7041 diets, only 17 had errors. Of these, almost two-thirds were critical. Majority of errors took place during diet card preparation (52.94%), by dietitians (70.59%), during weekdays (82.35%), breakfasts (47.06%), and in the cardiac care ward (47.06%). The causes identified through interviews were lack of backup or accessory food staff, and employee's personal and domestic issues. It was concluded that though the prevalence of dietary errors was low in this study, critical errors constituted majority of these errors, which may hamper clinical condition of patient. Adopting organizational behavior principles in hospital, by considering human factors, may not only reduce dietary errors, but improve patient well-being, and employee satisfaction in a long run.

Introduction

Healthcare organizations strive to deliver quality and safe healthcare to patients to ensure optimum treatment outcome which reflects the capability of the hospital and its staff (1). This is why patient safety is one of the important components identified by Institute of Medicine (IOM) and World Health Organization that outline principles for the healthcare organizations to provide quality healthcare to the patients (2), thus minimizing preventable harm and reducing risks during the delivery of healthcare (3,4). It is estimated that 10% of patients in high income countries face preventable adverse events during their hospital stay (4). In low-middle income countries, the adverse health events during hospital stay result in 2.6 million deaths annually (4). Dietary errors are one of the main preventable risks to patient safety (5–7). According to a study in Australia, 8% dietary errors were recorded in the hospital's electronic food delivery system (5). Another similar study reported that about 20% of the therapeutic meals served to in-patients in a metropolitan tertiary hospital, were inaccurate and 64.8% of these errors were critical in nature that could claim the life of a patient (7).

A therapeutic diet is one of the essential components of latest clinical treatment. Patients are prescribed these diets to cope with and/or recover their health while being admitted in hospital (8,9). Therefore, food and nutrition play a key role in recovery of patients (10). On the other hand, provision and consumption of inaccurate therapeutic diet could interfere with the patients' treatment and may pose a threat to their wellbeing (8,9,11).

The computerized system has shown to minimize errors in the hospitals' meal delivery process (5). However, errors are inevitable even in the computerized systems as these systems are operated by humans and to err is human. This study reports an account on the accuracy of meals delivered to in-patients, needing a therapeutic diet, admitted in one of the top international for-

profit hospitals with strictly implemented American hospital standards. We aim to assess the prevalence of errors in the provision of therapeutic diets to in-patients, and to explore the underlying causes to suggest strategies to reduce the chances of such errors. We expect that this study will serve as a guide to enhance patient safety, improve patient satisfaction, and effectiveness of the meal delivery systems in international level hospitals located in low-middle income countries.

Methods

We employed a sequential explanatory mixed-methods study design from 1st March 2019 to 31st March 2019. This design entails a quantitative approach followed by qualitative inquiry to explain the quantitative findings and to enhance the utility of findings for the concerned quarters (12). We collected quantitative data by observing all meals that were provided to the inpatients for one month. For qualitative data, we interviewed the sources of errors- the respective staff in the food department of the hospital. The study tool used for this study could be accessed in the supporting document 1 titled “Study Tool”. The ethical permission to conduct this study was granted by the Advance Studies & Review Board of the Khyber Medical University, Pakistan (No. DIR/KMU-AS&RB/IE/000966 dated 17th January 2020). The study was conducted in a private hospital, located in the capital of Pakistan.

The food preparation and its delivery to the patients involved different sections of the food department in the hospital. The relevant clinical staff recommended the diet of the inpatients in their clinical notes, based on patient’s need. The respective unit representative of the ward entered the diet in the computer system with patient details. The computer system then

generated requests to the hospital food department for food preparation as per provided requirements.

There were two ways of tracking the errors in the system (Tables 1 & 2). When an error was noticed (Table 1), the source of error was tracked and identified and the inaccurate diet was replaced with the correct one. For the errors outlined in Table 2, the error could not be detected unless the patient developed symptoms following consumption of inaccurate diet, which was then checked with biochemical analysis to establish causality. There were a total of five check points in the system and dietary errors could occur at any of these five points:

1) Diet entry

   Diet entry was the first point where patient's diet was entered the system by the Unit Representative (UR) and was the primary source of information provided to the dietary staff.

2) Diet card preparation:

   A hard copy of detailed diet information was then forwarded to the dietitian who made diet cards based on the information provided.

3) Meal packing & tagging:

   The kitchen received requests for food through the diet card. The kitchen personnel packed and tagged food according to the diet card, for example, renal diet, no salt or no chili diet, etc.

4) Tray preparation:

The tray was prepared according to the diet card and cross checked by the dietician who tallied the diet card with tag on the meal placed in the tray ready to be served to patient.

5) Meal provision:

The checked diet was then carried out in a heated trolley to the patient room. Before serving it to the patient, it was again cross checked by the floor staff with patient notes. If an error was detected, it was immediately reported to the food department for replacement. In case the error went unnoticed and the patient consumed the diet, the later vital statistics of the patient might indicate the incorrect diet.

Table 1: Classification of Dietary Errors as per Hospital's Criteria before the Consumption of Meals

**Advised Diet**	**Received Diet**	**Error Type**
Regular	Soft/Full Liquid/ Semi Solid/Clear Liquid	Non-Critical Error
Soft	Full Liquid/ Semi Solid/Clear Liquid	Non-Critical Error
Semi Solid	Full Liquid/Clear Liquid	Non-Critical Error
Full Liquid	Clear Liquid	Non-Critical Error
Semi Solid	Regular/ Soft	Critical Error
Full Liquid	Regular/ Soft/ Semi Solid	Critical Error
Clear Liquid	Regular/Soft/Full Liquid/ Semi Solid	Critical Error
Allergic Patient	Received Allergens	Critical Error
Patient on intolerance Diet	Received any item that cause indigestion	Critical Error
NPO (Nil per Oral)	Received food item	Critical Error
Neutropenic Diet patient	Received raw/ uncooked item	Critical Error

Table 2: Classification of Dietary Errors as per Biochemical Reports after Wrong Meal Consumption According to Hospital's Criteria

Recommended Patient Diet	TEST	Critical Values	Error
**ADA**	Blood Glucose Level	380mg/dl (Type II),<br>500mg/dl (Type I)	Critical
**Low salt**	Blood Pressure	180/110mmHg	Non-Critical
**No salt**	Blood Pressure	210/110mmHg	Critical
**Low Cholesterol**	Lipid Profile	More than 210mg/dl	Non-Critical
**No Cholesterol**	Lipid Profile	>240mg/dl	Critical
**Renal**	Chem 7	6.5mEq/L	Critical

Census sampling technique was used for the collection of quantitative data over a period of one month (March 2019). The dietary information of patients admitted for at least 24 hours to any of the seven wards (medical, cardiac care unit, kidney transplant, gastroenterology, orthopedics, surgical, and neurology) of the hospital during the study period was included in the study. The dietary information from the comatose patients, and patients on nasogastric feed was excluded from the study. For in-depth interviews (IDIs), purposive sampling technique was used. After detection of the point of error, the person responsible for error was explained the purpose of the interview/research, and subsequently orally consented for interview(s), to seek the causes and reasons of the error.

We collected qua[illegible]tive data by observing all meals provided to inpatients on a structured proforma, designed based on the variables that were either available and/or could be collected from the hospital system. The outcome variable was "diet error", a binary variable with two possible outcomes: Critical / Non-Critical Errors. The definitions for critical/non-critical errors were based on the definitions provided by the hospital (Tables 1 & 2).

There were a total of five independent variables in this study, all categorical in nature. The accuracy of all the therapeutic meals were assessed at five main points in the food delivery system namely “point of error”: (1) Diet entry; (2) Diet card making; (3) Meal packing; (4) Tray preparation; and (5) Meal provision. The “responsible staff” included the unit representative, dietitian, cook, and service aide. The “Day of the Week” was a binary variable, defined as ‘Weekday’ and ‘Weekend’. The “Meal Type” included breakfast, lunch, and dinner. The “Ward” variable was composed of five levels: renal, cardiac, gastroenterology, orthopedics, and surgical wards.

All the quantitative variables were categorical in nature. The row percentages were calculated for all the independent variables against the outcome variable. We calculated frequencies and percentages for all the critical and non-critical errors using SPSS version 25 (13).

The IDIs were conducted in the local language, audio recorded, transcribed, and translated into English language. To facilitate the interview process, a semi-structured interview guide was developed, based on the consensus of the authors. A total of three questions were prepared as a start. The same questions were asked to everyone. Each interview lasted for up to 30 minutes maximum.

The qualitative data was collected, through in-depth interviews using semi-structured questionnaire from the person responsible for dietary error, till saturation of data was achieved to explore all the possible reasons of dietary errors. The interviews were analyzed using a combined deductive-inductive thematic analysis approach (14). The IDIs were transcribed by two researchers with cross-checking, discussion on notes and comparing them with audio

recordings. The same researchers coded the data from transcripts. Codes with similar concepts were grouped into explicit themes.

Resul[illegible]

A total of 7041 meals were observed including breakfast, lunch, and dinner of all the in-patients during the study period. Of all the meals with dietary errors, 64.71% were critical and 35.29% were non-critical in nature (Table 3). Majority of the total dietary errors occurred during the diet card preparation (52.94%), by the dietitians (70.59%), during weekdays (82.35%), at breakfast time (47.06%), and in the cardiac care unit (47.06%).

Table 3: The Percentage Wise Distribution of Dietary Errors (N=7041)

**Variables**		**Critical Errors N (%)**	**Non-Critical Errors N (%)**	**Total Errors N (%)**
**Overall**		11 (64.7)	6 (35.3)	17 (0.24)
**Point of Error**	Diet Card Preparation	7 (77.8)	2 (22.2)	9 (52.9)
	Diet Entry	1 (33.3)	2 (66.7)	3 (17.7)
	Packing	0 (0)	1 (100)	1 (5.9)
	Tray line	2 (66.7)	1 (33.3)	3 (17.7)
	Meal Provision	1 (100)	0 (0)	1 (5.9)
**Responsible Staff**	Dietitian	9 (75.0)	3 (25.0)	12 (70.6)
	Unit Representative	1 (33.3)	2 (66.7)	3 (17.6)
	Cook	0 (0)	1 (100)	1 (5.9)
	Service Aide	1 (100)	0 (0)	1 (5.9)
**Weekday**	Yes	10 (71.4)	4 (28.6)	14 (82.3)
	No	1 (33.3)	2 (66.7)	3 (17.7)
**Meal Type**	Breakfast	5 (62.5)	3 (37.5)	8 (47.0)

	Lunch	4 (66.7)	2 (33.3)	6 (35.3)
	Dinner	2 (66.7)	1 (33.3)	3 (17.7)
**Ward**	Renal Transplant	2 (66.7)	1 (33.3)	3 (17.7)
	Cardiac Care	7 (87.5)	1 (12.5)	8 (47.1)
	Gastroenterology	1 (50.0)	1 (50.0)	2 (11.8)
	Orthopedics	0 (0)	3 (100)	3 (17.7)
	Surgical	0 (0)	1 (100)	1 (5.9)

As critical dietary errors may risk the life of under-treatment patient, we made a comparison between critical and non-critical dietary errors at each level of the diet preparation and delivery process in Table 3. We observed that majority of the critical errors took place during tray preparations (66.67%), meal provision (100%), diet card preparations (77.78%), by the dietitians (75.00%), service aides (100%), in all the meal types (~66%), in renal transplant (66.67%) and cardiac care unit (87.50%).

We interviewed a total of 11 staff members responsible for dietary errors, after which a saturation in responses was received. Some of the staff in the food department attributed the dietary errors to the increased workload while others could not concentrate on work due to various reasons. The themes that emerged from the analysis of the interviews are as follo[illegible]

The participants stated that hospital had limited number of employees in the food department and did not have a backup staff to replace any staff member on leave. One of the employees attributed the reason of dietary errors to the inability to handle increased demand load due to increased admissions. The food staff responsible for the diet error said;

"The patient load was too high for me to handle single-handedly". (A male in his 20's)

Similarly, a food staff came back after a long leave and could not handle patient load on the first few days;

> *"I was feeling tired as the patient load was too much. I could not concentrate after a long break".* (A male in his 20's)

The lack of concentration, in some instances, was attributed to increased workload by the participants especially in cases where rare situations were encountered which resulted in critical errors. Rare situations were reported as food allergies of some patients or presence of two patients with exact same names but different rooms.

> *"I didn't notice the allergy note as it was not prominent. There are so many patients' diet to handle, so it went unnoticed".* (A female in her 20's)

> *"The patient name was same and I didn't pay attention to different room numbers. That day there were lot of food trays for delivery to patients. I should have checked the names and room numbers before serving anything to patients".* (A male in his 40's)

According to participants, burnout due to working overtime to compensate for absent staff led to increased chances of errors. One of the food staff mentioned that;

> *"Sometimes we need to perform extra shift to cover for the staff on leave, this becomes burden and tiresome as the working hours become too lengthy".* (A male in his 20's)

Another explicitly added;

> *"The management does not have a backup staff to cover for staff on leave that may get absent for any reason including getting sick".* (A male in his 30's)

Another theme that emerged was the employees' personal and domestic issues because of which they were unable to concentrate at work. Some of the employees attributed the reasons for dietary error to distributed concentration at work as a result of incidents at home. For instance, one of the staff mentioned;

"I was stressed as my spouse got injured in a road traffic accident, I needed a leave but could not get it, and therefore, I could not focus at work". (A female in her 20's)

Another expressed a job related pressure from home,

"My spouse wants me to leave the job and be at home". (A female in her 20's)

Another participant had an exchange of words at home as a result of which the staff was depressed;

"I had an argument with mother-in-law just few minutes before leaving home for duty. This made me depressed and I could not pay attention at work". (A female in her 20's)

Similarly, one of the food staff mentioned being unable to get leave due to illness as a reason for dietary errors;

"I didn't sleep well just because I was not feeling well, so I was disturbed during my duty or you can say I was absent minded". (A female in her 20's)

Discussio[illegible]

Nutrition therapy is often used in conjunction with the medical treatment to support and optimize the overall treatment outcome of the hospitalized patients (15). The delivery of correct therapeutic diet is of paramount importance, in this regard. The prevalence of critical dietary errors accounted for majority of the dietary errors in our study. This result is consistent with other observational studies where the proportion of critical errors was the highest (5,7). The majority of dietary errors took place during the diet card preparation followed by diet entry into the computerized system and the tray line. These findings are comparable to a study conducted in Thailand, which also related majority of the dietary errors to diet card preparation in the diet flow process (16). The diet entry and diet card preparation processes are crucial being the initial steps in the diet flow process. It is imperative that these steps remain error free to prevent the

error carried all the way to the patient. The main reasons of dietary errors were the lack of back up staff and the employees' personal and domestic issues in this study.

The dietitians' had two tasks at hand: to prepare diet cards and to check the diets with prepared diet cards during the tray preparation. In this study, about 70% of errors were attributed to the dietitians. This result is congruent to the Australian study where dietitians made up for majority of dietary errors (5). Manual diet card preparation is not only cumbersome but also increases chance of error in case of increased workload. The human error can be prevented by getting the diet cards printed via computer systems or through automation as soon as the unit representative of the ward inputs the diet information into the computerized system (17). Previous studies reported a significant reduction in dietary errors after introduction of automated diet cards' preparation in hospital settings (5,7,18). To have error free diet cards, it is imperative to have error free diet entry into the computerized system by the unit representative. The error free diet entry in the system could be ensured by double entry computerized systems as evident from research (19). Alternatively, a tablet or phone based e-application could be developed, with a digital checklist diet form, linked to the patient's electronic record system to prevent dietary errors (17). Any diet entry that conflicts with the medical condition of the patient would generate an error upon incorrect diet selection. This system may have upfront cost but in the long run, would save expenses related to diet replacements, time, cooks' labor, and medical expenses in case of inaccurate diet consumption. It is noteworthy that none of the dietary errors were attributed to the floor staff. This may indicate that a visual check of the diet with patient notes is a fruitful process of detecting dietary errors.

The reduction of in-patient dietary errors is of highest priority to improve the quality of patient care and treatment outcome. Humans are emotional beings (20) and any factor associated with their emotions may affect their work performance (21). The common reasons for the errors were related to human capacity and human needs, e.g. increased workload, personal and domestic issues, limited capacity to manage patient load, and overwork burn out. There was less probability that someone would be available at home over the weekdays to take care of emergency issues, if one arises like narrated by the participants in this study. Similarly, start of the day may not be good if the employee's concentration is not over the designated tasks (22,23).

The private for-profit hospitals in low middle-income countries, like Pakistan, work on limited resources to maximize profits. In such countries, technology is expensive as compared to human resource. The findings of this study suggest that human errors could be minimized by adapting organizational behavior skills. For example, integrating empathy with the human resource management to improve employee job satisfaction. To do this, there should be a manageable workload on the employee. On-job training should be provided for capacity development of the food staff, and availability of back-up staff at all times to cater for increased patient load or replacement for staff on leave, so as not to compromise on quality and safety to in-patients.

The findings of this study come with a limitation on generalizability as the study was conducted in one hospital. However, this was the only hospital in the country that had implemented international standards and provided therapeutic dietary meals to in-patients as a part of treatment regimen. The study derives its strength from the large number of observations. Additional strength is the qualitative component of the study which explored actual causes of

dietary errors in the meal flow process. This is a firm strength of the study as qualitative components have not been explored in any of the earlier studies.

Conclusio[illegible]

The critical dietary errors formed the highest concern in the hospital food department in this study. This is alarming as such errors could claim the life of a patient. It is noteworthy that no deaths were reported in this study as a result of dietary errors. The major reasons for these errors were attributed to human factors associated with basic human needs for which the hospital may be required to ensure and adhere to the basic principles of organizational behavior for contented employees and error-free therapeutic food services to the patients for patient safety, satisfaction, and well-being.

References

1. Zhang X, Hauck K, Zhao X. Patient safety in hospitals - a Bayesian analysis of unobservable hospital and specialty level risk factors. Health Econ. 2013 Sep;22(9):1158–74.
2. Baker A. Crossing the quality chasm: a new health system for the 21st century. Vol. 323. British Medical Journal Publishing Group; 2001.
3. World Health Organization. The world health report: health systems financing: the path to universal coverage: executive summary [Internet]. World Health Organization; 2010. Available from: https://www.who.int/whr/2010/en/
4. Stiefel M, Nolan K. A guide to measuring the triple aim: population health, experience of care, and per capita cost. IHI innovation series white paper Cambridge, Massachusetts: Institute for Healthcare Improvement. 2012;
5. Rattray M, Desbrow B, Roberts S. Identifying errors in meals provided to and sourced by patients on therapeutic diets in hospital. Asia Pac J Clin Nutr. 2018;27(3):533–9.
6. Rattray M, Desbrow B, Roberts S. Comparing nutritional requirements, provision and intakes among patients prescribed therapeutic diets in hospital: An observational study. Nutrition. 2017 Aug;39–40:50–6.
7. Larby A, Roberts S, Desbrow B. Accuracy and adequacy of food supplied in therapeutic diets to hospitalized patients: An observational study. Nutrition & Dietetics. 2016;73(4):342–7.
8. Dietitians Association of Australia, Speech Pathology Association of Australia Limited. Texture-modified foods and thickened fluids as used for individuals with dysphagia: Australian standardised labels and definitions. Nutrition & dietetics. 2007;64:S53–76.
9. Sicherer SH, Sampson HA. Food allergy. J Allergy Clin Immunol. 2010 Feb;125(2 Suppl 2):S116-125.
10. Niedert KC, American Dietetic Association. Position of the American Dietetic Association: Liberalization of the diet prescription improves quality of life for older adults in long-term care. J Am Diet Assoc. 2005 Dec;105(12):1955–65.
11. Carmont MR. The Advanced Trauma Life Support course: a history of its development and review of related literature. Postgrad Med J. 2005 Feb;81(952):87–91.
12. Schoonenboom J, Johnson RB. How to construct a mixed methods research design. KZfSS Kölner Zeitschrift für Soziologie und Sozialpsychologie. 2017;69(2):107–31.
13. IBM Corp. IBM SPSS Statistics for Windows. Armonk, NY: IBM Corp.; 2017.
14. Braun V, Clarke V. Using thematic analysis in psychology. Qualitative research in psychology. 2006;3(2):77–101.

15. Barker LA, Gout BS, Crowe TC. Hospital malnutrition: prevalence, identification and impact on patients and the healthcare system. Int J Environ Res Public Health. 2011;8(2):514–27.
16. Tonjang S, Thawesaengskulthai N. Patient Food Delivery Error in the Hospital: A Case Study in Thailand. In 2019. p. 151–6.
17. Paulsen A, Overgaard S, Lauritsen JM. Quality of Data Entry Using Single Entry, Double Entry and Automated Forms Processing–An Example Based on a Study of Patient-Reported Outcomes. PLOS ONE. 2012 Apr 6;7(4):e35087.
18. Zhao C. Factors influencing trayline accuracy at long-term care facilities that use tray menus and disposable tray cards [Internet]. 2001. Available from: https://twu-ir.tdl.org/handle/11274/10036
19. Kawado M, Hinotsu S, Matsuyama Y, Yamaguchi T, Hashimoto S, Ohashi Y. A comparison of error detection rates between the reading aloud method and the double data entry method. Controlled clinical trials. 2003;24(5):560–9.
20. Panksepp J. Affective consciousness: Core emotional feelings in animals and humans. Consciousness and Cognition. 2005 Mar 1;14(1):30–80.
21. Quoidbach J, Hansenne M. The Impact of Trait Emotional Intelligence on Nursing Team Performance and Cohesiveness. Journal of Professional Nursing. 2009 Jan 1;25(1):23–9.
22. de Mello MT, Esteves AM, Pires MLN, Santos DC, Bittencourt LRA, Silva RS, et al. Relationship between Brazilian airline pilot errors and time of day. Brazilian Journal of Medical and Biological Research. 2008;41(12):1129–31.
23. Kozer E, Scolnik D, Macpherson A, Keays T, Shi K, Luk T, et al. Variables Associated With Medication Errors in Pediatric Emergency Medicine. Pediatrics. 2002 Oct 1;110(4):737–42.

Supporting Document 1: Study Tool

Click here to access/download
Supporting Information
Questionnaire.docx
