# Supplementary material for: The importance of human factors in therapeutic dietary errors of a hospital: A mixed-methods study
Source: PLoS One. 2022 Aug 25;17(8):e0273728. doi: 10.1371/journal.pone.0273728 (PMC9409594; doi:10.1371/journal.pone.0273728)
Supplement: Attachment — Submitted filename: PLOS ONE paper review 8.docx [file pone.0273728.s003.docx]

**PONE-D-21-39046**

**Re: “The importance of human factors in therapeutic dietary errors of a hospital: A mixed methods study”**

Thank you for asking me to review this manuscript. I have the following **Comments:**

**Abstract:** It should be rewritten in a structure format as per PLOS journal guidelines.

**Introduction:**

The authors quoted 11 references under introduction with only three short paragraphs. The introduction will benefit from details explanation related to the objectives of this study. Start with the definition of dietary errors and its examples.

Consider replacing “err” on line 71 by “error”.

**Methods:**

This section requires major revision.

The study tool used for this study on line 87-88, should be referenced or accessed through a link.

The validity of questionnaire and their Cronbach’s alpha and reliability scores should be reported after data collection.

Explain in more details’ inclusion and exclusion criteria of the study.

How were the data extracted? What were the sampling technique? What was the sampling equation to calculate sample size? How did the authors come up with 7041?

Explain in detail the independents variables?

**Qualitative study**:

The one conducting interview should have experience in qualitative research.

Experts in qualitative research should supervised data collection and hold feedback sessions shortly after the interview.

All interviews should be recorded and transcribed in full to verify the accuracy of responses.

Two members of the research team independently should analyze the transcribed responses and read them on multiple times to familiarize self with the contents and categorize it in a meaningful way.

All these points should be explained and incorporated under the design of qualitative study.

**Quantitative study:**

It should be explained in details and the collected data should be reported. Authors should do statistical analysis for quantitative data.

The authors stated on lines 144-145 that “The dietary information from the comatose patients, and patients on nasogastric feed were excluded from the study” Why? No patients interview was carried out in this study.

What was the definition of dietary errors in this study?

Table 1 and 2 should be moved under results.

What was ADA in Table 2 stand for?

**Ethical consideration:** needs to be under subheading after methods. There were issues with confidentiality and power imbalance with the recruitment process and I was unsure what the recruitment process was?

**Results:** Should be rewritten once the methods revised.

Statistical analysis should be done and report p-values. Furthermore, relationship between dependent and independent variables should be done from statistical analysis point of view.

Explain in details the personal and familial issues of the participants in relation to dietary errors.

**Conclusion:** It should be re-written with a major focus on the main results of the study. In addition, one statement of recommendation based on the findings of the study should be mentioned under conclusion. The statement about deaths in this study (lines 315-316), should be deleted as this was not investigated in this study.

**Limitations of the study:** should explain in details and logical ways. Personal, recall, misinterpretation biases and Hawthorne effect were possibilities in this study

**References:** The manuscript presents several references which were outdated, and some were irrelevant. The references should be revised, updated, and only used if it were relevant to the study. It also should follow the guidelines of the journal.
