# Supplementary material for: The importance of human factors in therapeutic dietary errors of a hospital: A mixed-methods study
Source: PLoS One. 2022 Aug 25;17(8):e0273728. doi: 10.1371/journal.pone.0273728 (PMC9409594; doi:10.1371/journal.pone.0273728)
Supplement: Attachment — Submitted filename: Response to Reviewers.docx [file pone.0273728.s005.docx]

**July 05, 2022**

**Yong-Hong Kuo**

Academic Editor, PLOS ONE

**Subject: Response to Reviewers’ Comments**

Honorable Yong-Hong Kuo,

We thank the reviewers for taking out precious time to review our manuscript. The comments serve to shape the manuscript to its perfection. Thank you for the constructive feedback on our manuscript, which we have carefully incorporated in the revised manuscript.

Please find responses to each comment below.

Thank you very much.

**Response to Journal Requirements:**

Please review your reference list to ensure that it is complete and correct. If you have cited papers that have been retracted, please include the rationale for doing so in the manuscript text, or remove these references and replace them with relevant current references. Any changes to the reference list should be mentioned in the rebuttal letter that accompanies your revised manuscript. If you need to cite a retracted article, indicate the article’s retracted status in the References list and also include a citation and full reference for the retraction notice.

**Response: To our knowledge, none of the papers cited have been retracted. We double checked all the articles for retraction. However, the reference number 4 had an old link that was changed after the submission of the manuscript by the publishers. The link has been updated now for the paper referenced as number 4. Thank you for noticing this.**

**P.S: The referenced article 4 does not depict tracked changes in the “references” section for some reasons.**

**Response to Reviewer # 4:**

Thank you very much for allowing me to review this manuscript for the second time.

Most of the required corrections have been made and the manuscript had been improved scientifically. The authors responded to majority of my comments and provided valuable information which are important for the readers. I further have the followings minor comments:

1. The validity of questionnaire and their Cronbach’s alpha and reliability scores should be reported after data collection. This was communicated to the authors, however they felt that this do not apply for both quantitative and qualitative components of the study as the questionnaire was not meant to conduct a survey in the field. Regardless, as long as the questionnaire was used in this study, its validity and their Cronbach’s alpha and reliability scores should be calculated.

**Response: The questionnaire was formed to assist the student researcher to extract data from secondary sources (hospital records) so that only that information is gathered that is related to the research question and to prevent any deviation or distraction for the student researcher. It should be noted that neither latent variables were constructed nor any field survey was conducted in our study. Also, the questionnaire did not use any Likert scale. The honorable reviewer remarks that as the word "questionnaire" has been used, therefore, Cronbach's alpha should be applied regardless. We tried to get more information on this topic by looking at the literature and realized this doesn't apply to our research. Please follow the articles below that justify our claim:**

1. **Making sense of Cronbach's alpha**

[**https://www.ncbi.nlm.nih.gov/pmc/articles/PMC4205511/**](https://www.ncbi.nlm.nih.gov/pmc/articles/PMC4205511/)

2. **The Use of Cronbach’s Alpha When Developing and Reporting Research Instruments in Science Education**

[**https://link.springer.com/article/10.1007/s11165-016-9602-2**](https://link.springer.com/article/10.1007/s11165-016-9602-2)

3. **Cronbach Alpha Coefficient**

[**https://www.sciencedirect.com/topics/nursing-and-health-professions/cronbach-alpha-coefficient**](https://www.sciencedirect.com/topics/nursing-and-health-professions/cronbach-alpha-coefficient)

4. **Using and Interpreting Cronbach’s Alpha**

[**https://data.library.virginia.edu/using-and-interpreting-cronbachs-alpha/**](https://data.library.virginia.edu/using-and-interpreting-cronbachs-alpha/)

5. **Cronbach’s Alpha: Definition, Interpretation, SPSS**

[**https://www.statisticshowto.com/probability-and-statistics/statistics-definitions/cronbachs-alpha-spss/**](https://www.statisticshowto.com/probability-and-statistics/statistics-definitions/cronbachs-alpha-spss/)

6. **Cronbach’s Alpha and Semantic Overlap Between Items: A Proposed Correction and Tests of Significance**

[**https://www.ncbi.nlm.nih.gov/pmc/articles/PMC8867700/**](https://www.ncbi.nlm.nih.gov/pmc/articles/PMC8867700/)

**If the concern is over the word “questionnaire”, we can rephrase the manuscript that the data were drawn from secondary sources as an alternative, skipping the term questionnaire at all. We wrote the manuscript in complete honesty. This is why we explained everything in detail in addition to allow for reproducibility of the research. However, to address the concern of the reviewer in this scenario, we have added the issue of Cronbach’s alpha in the limitations section of our manuscript, which could be found at page 17 lines 365-370.**

2. Authors should do statistical analysis for quantitative data. Furthermore, relationship between dependent and independent variables should be done from statistical analysis point of view. The authors responded to this issue as the objective of the study was neither to explore the associations nor the factors associated with the outcome variable nor to test a hypothesis, therefore, inferential statistics were not deployed, rather only descriptive statistics were reported. I do not think this is the case as further statistical analysis will help to achieve the objectives of the study and use independent variables in this study to predict the value of dependent variable (dietary errors). I think such statistical analysis will strengthen the findings of this study.

**Response: Majority of the times, inferential statistics are used when one is testing a hypothesis or wants to explore the associations. Our study did not have any such objectives. We used only descriptive statistics because a critical error in the hospital setting could hamper a patient's life, regardless if it's statistically significant or not. In our expertise, the use of inferential statistics doesn't justify in this manuscript as the factors associated with the errors have been explored in the qualitative component of the manuscript.**

**However, in the light of making the manuscript a bit more scientific as per reviewer’s comment, we applied the inferential statistics as it would not jeopardize the study objectives. The Fisher-exact based p-values have been reported to the Table 3 on pages 10-11. A bit of the description has also been added to the text on page 9, lines 187-189 and page 11, lines 231-232. Thank you.**
